# A *Salmonella* Toxin Promotes Persister Formation through Acetylation of tRNA

**DOI:** 10.1016/j.molcel.2016.05.002

**Published:** 2016-07-07

**Authors:** Angela M. Cheverton, Bridget Gollan, Michal Przydacz, Chi T. Wong, Anastasia Mylona, Stephen A. Hare, Sophie Helaine

**Affiliations:** 1 Section of Microbiology, Medical Research Council Centre for Molecular Bacteriology and Infection, Imperial College London, London SW7 2AZ, UK; 2 Department of Life Sciences, Imperial College London, London SW7 2AZ, UK

## Abstract

The recalcitrance of many bacterial infections to antibiotic treatment is thought to be due to the presence of persisters that are non-growing, antibiotic-insensitive cells. Eventually, persisters resume growth, accounting for relapses of infection. *Salmonella* is an important pathogen that causes disease through its ability to survive inside macrophages. After macrophage phagocytosis, a significant proportion of the *Salmonella* population forms non-growing persisters through the action of toxin-antitoxin modules. Here we reveal that one such toxin, TacT, is an acetyltransferase that blocks the primary amine group of amino acids on charged tRNA molecules, thereby inhibiting translation and promoting persister formation. Furthermore, we report the crystal structure of TacT and note unique structural features, including two positively charged surface patches that are essential for toxicity. Finally, we identify a detoxifying mechanism in *Salmonella* wherein peptidyl-tRNA hydrolase counteracts TacT-dependent growth arrest, explaining how bacterial persisters can resume growth.

## Highlights

• TacT promotes *Salmonella* persister formation by inhibiting translation
• TacT is an acetyltransferase with positively charged patches essential for toxicity
• TacT blocks the primary amine group of amino acids on charged tRNA molecules
• *Salmonella* detoxifies TacT-corrupted tRNAs, allowing bacterial growth to resume

## Graphical Abstract

**Figure:**
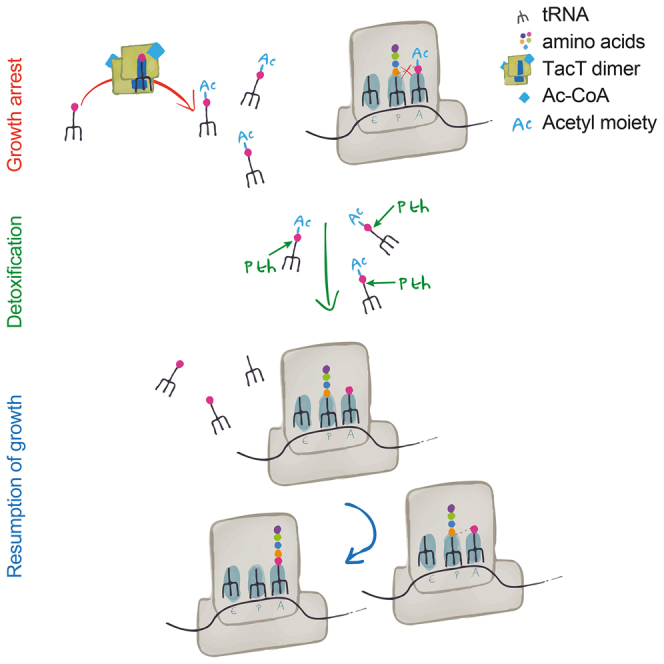

## Teaser

Persisters are non-growing, antibiotic-insensitive bacteria involved in recalcitrance of infections. *Salmonella* forms intra-macrophage persisters through the action of toxin-antitoxin modules. Cheverton et al. reveal that TacT is an acetyltransferase toxin blocking the primary amine of amino acids on charged tRNAs. They identify a detoxifying mechanism counteracting TacT-dependent growth arrest, allowing growth resumption.

## Introduction

Chronic and relapsing infections are a major problem to human health as such infections can cause considerable morbidity and frequently require multiple courses of antibiotics, which in turn are thought to contribute to the emergence of stable antibiotic resistance. Such long-lasting infections are caused by a variety of bacteria, including *Mycobacterium tuberculosis*, *Salmonella*, *Pseudomonas*, pathogenic *Escherichia coli*, *Staphylococcus*, and *Streptococcus* species. The recalcitrance of these infections to antibiotic treatment is thought to be due, at least in part, to the presence of persister cells (Helaine and Kugelberg, 2014, Mulcahy et al., 2010) that are multi-drug tolerant. Persisters have not acquired mutations conferring resistance but undergo a transient phenotypic switch and enter a non-growing state in which they are not affected by antibiotics (Balaban et al., 2004). It is thought that eventually persisters can resume growth, accounting for relapses of infection. In humans, *Salmonella enterica* serovar Typhimurium (*S*. Typhimurium) causes gastroenteritis and more invasive disease in HIV- or malaria-infected patients, who can also suffer relapse after antibiotic treatment (Okoro et al., 2012). *S*. Typhimurium causes disease by proliferating inside mammalian cells, including macrophages.

Using a fluorescence-based method (fluorescence dilution) for direct quantification of the dynamics of intracellular replication of bacteria at the single-cell level (Helaine et al., 2010), we reported that after macrophage phagocytosis, a significant proportion of the *Salmonella* population ceases to grow, while remaining metabolically active (Helaine et al., 2010), and survives antibiotic treatment (Helaine et al., 2014). A family of genes encoding class II toxin-antitoxin (TA) modules is involved in the induction of *E. coli* persisters during growth in laboratory medium (Maisonneuve et al., 2011). We found that a repertoire of class II TA modules in *Salmonella* induces the formation of intracellular persisters (Helaine et al., 2014). Class II TA operons encode a non-secreted toxin, which inhibits an essential cellular function such as RNA translation or DNA replication, and an antitoxin, which interacts with and neutralizes the toxin (Yamaguchi and Inouye, 2011). The toxin is relatively stable, whereas the antitoxin is labile and degraded under various stress conditions, leading to a buildup of free toxin and growth arrest of the bacterial cell (Yamaguchi and Inouye, 2011). More generally, growth arrest is an important but overlooked aspect of *Salmonella* virulence and probably of other bacterial pathogens. Little is known about the molecular mechanisms of *Salmonella* toxin activity, persister induction, and growth resumption.

Bioinformatics analysis of the *S.* Typhimurium genome sequence revealed the presence of at least 14 putative class II TA modules (De la Cruz et al., 2013, Helaine et al., 2014, Lobato-Márquez et al., 2015, Shao et al., 2011), of which only one, VapC, has been functionally characterized (Winther and Gerdes, 2009, Winther and Gerdes, 2011). We showed by mutational analysis that each of the 14 loci contributes to intramacrophage persister formation (Helaine et al., 2014). Eleven of the TA modules can be classified into four known TA families based on significant amino acid sequence identity with known toxins of *E. coli* (Yamaguchi and Inouye, 2011). The other three *Salmonella* TA modules belong to a previously uncharacterized family, in which the toxin shares amino acid similarity with Gcn5 *N*-acetyltransferases (GNATs). GNAT superfamily enzymes catalyze acetyl group transfer from acetyl-coenzyme A (Ac-CoA) to a wide range of substrates (Hentchel and Escalante-Semerena, 2015). By investigating the activity and target of one of these GNAT toxins, T8, we reveal that this toxin governs *Salmonella* entry into the persister state through acetylation of aminoacyl-tRNA molecules, thereby halting translation. We characterized the structural and mechanistic basis of this activity. We also identified a detoxifying process that allows the bacteria to exit the growth-arrested state and resume proliferation. This might open up routes to force bacteria out of their persister state and become susceptible to antibiotic treatment.

## Results

### A GNAT-Related Toxin of *Salmonella* Extends Lag Phase by Halting Protein Synthesis

**Figure 1:**
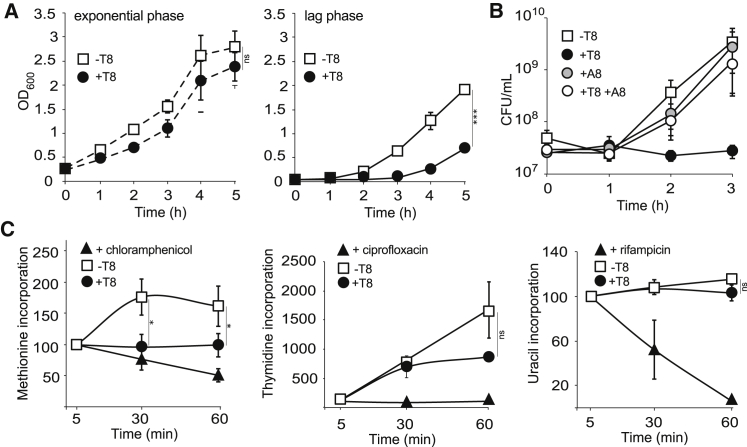
A GNAT-Related Toxin of *Salmonella* Extends Lag Phase by Halting Protein Synthesis (A) Growth curves of *S*. Typhimurium 12023 Δ*ta8* carrying pNDM220 (−T8) or pNDM220::*t8* (+T8) in fresh rich medium. Expression of *t8* was induced upon addition of IPTG during mid-exponential (left panel) or lag (right panel) phase. (B) Growth curves of *S*. Typhimurium 12023 Δ*ta8* carrying pNDM220 (−T8), pNDM220::*t8* (+T8), pBAD33::*a8* (+A8), or pNDM220::*t8* and pBAD33::*a8* (+T8+A8). All cultures were supplemented with arabinose and IPTG in fresh rich medium during lag phase. (C) Levels of incorporation of radiolabeled methionine (left panel), thymidine (middle panel), or uracil (right panel) during pulse-chase assays in lag phase cultures of *S*. Typhimurium 12023 Δ*ta8* carrying pNDM220 (−T8) or pNDM220::*t8* (+T8), or treated with bacteriostatic concentrations of chloramphenicol, ciprofloxacin, or rifampicin. All cultures were supplemented with IPTG at t0. All measures were normalized to those of the control samples at 5 min. Data represent the mean ± SEM (n ≥ 3) and were analyzed using a Student’s t test (ns, non-significant; ^∗^p < 0.05; ^∗∗∗^p < 0.005) (see also Figure S1).

Overexpression of the *t8* gene, encoding a GNAT-related putative toxin (Helaine et al., 2014), into a single deletion mutant of the corresponding TA module had little to no effect on *Salmonella* growth during the exponential phase (Figure 1A). However, when induced during lag phase, overexpression of *t8* extended the lag by several hours (Figures 1A and S1, available online), indicating that this toxin does not arrest growth per se, as described for other toxins, but rather locks the bacteria in a non-growth state. This growth inhibition was counteracted by concomitant overexpression of the corresponding antitoxin gene (Figures 1B and S1), showing that this operon encodes a bona fide TA module.

To determine the physiological process targeted by T8, we measured rates of DNA replication, mRNA transcription, and translation using pulse-chase of radiolabeled methionine, thymidine, or uracil (Winther and Gerdes, 2009). We treated a bacterial culture with chloramphenicol, ciprofloxacin, or rifampicin as positive controls. Upon induction of *t8* gene expression, methionine incorporation was immediately halted, whereas thymidine incorporation only declined 60 min after induction, and that of uracil remained unchanged (Figure 1C). These experiments indicate that T8 specifically blocks protein translation.

### T8 Is a Unique Acetyltransferase

**Figure 2:**
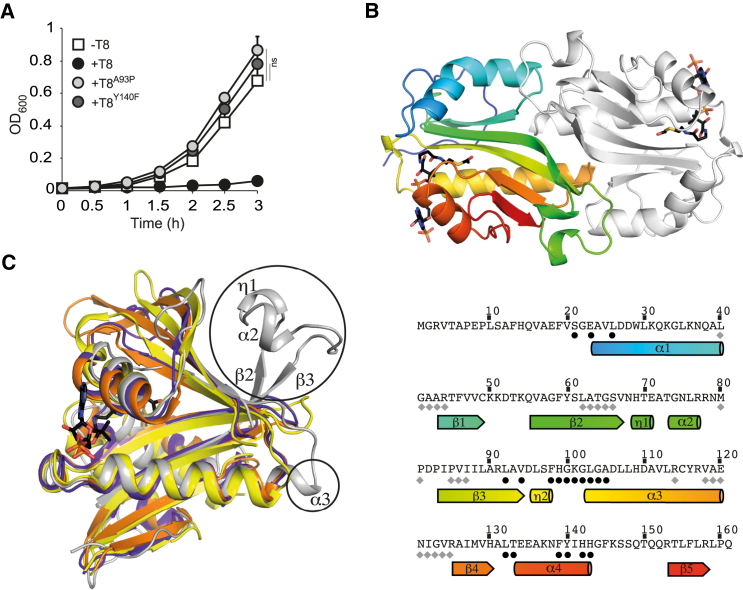
T8 Is a Unique Type of Acetyltransferase (A) Growth curves of *S*. Typhimurium 12023 Δ*ta8* expressing from pNDM220, the wild-type toxin (+T8), or point mutant toxins (+T8^A93P^, +T8^Y140F^), or carrying the empty vector (−T8). All cultures were supplemented with IPTG in fresh rich medium during lag phase. Data represent the mean ± SEM (n ≥ 3) and were analyzed using a Student’s t test (ns, non-significant). (B) Cartoon representation of the dimeric structure of T8^Y140F^ (top panel). Chain A is colored from blue at the N terminus to red at the C terminus, and Chain B is colored light gray. Ac-CoA molecules are shown as stick representations. Sequence of T8 (lower panel) with secondary structure elements illustrated and colored as in the top panel. Black dots indicate Ac-CoA binding residues and gray diamonds indicate residues involved in dimer formation (PDB: 5FVJ). (C) Superimposition of T8^Y140F^ (gray) with three other GNATs identified as being close structural homologs by the DALI server (Holm and Rosenström, 2010)—PDB: 2R7H (yellow), 2CNM (orange), and 4PV6 (purple), members of the N-acetyltransferase superfamily. Unique features of T8 are labeled and highlighted in black circles (see also Figures S2 and S3).

As GNATs had not been characterized previously as toxin components of TA modules, we questioned whether T8 toxicity relied on acetyltransferase activity. Acetylation is the transfer of an acetyl group from a donor, generally Ac-CoA, to a target molecule. GNATs in general share little amino acid identity (Hentchel and Escalante-Semerena, 2015); however, BLAST analysis of the T8 amino acid sequence indicated the presence of a conserved *N*-acetyltransferase superfamily domain, including an Ac-CoA binding pocket. We designed single amino acid substitutions of predicted Ac-CoA binding residues intending to block Ac-CoA binding (A93P) or to allow binding to Ac-CoA but prevent transfer of the acetyl moiety to the acceptor molecule (Y140F). Each substitution completely abolished T8 toxicity in *Salmonella* (Figure 2A).

The conserved Y140 natively coordinates an oxygen atom of Ac-CoA to ensure correct orientation of the acetyl group for transfer. Due to the conservative nature of the Y140F mutation, we predict that the only difference between T8^Y140F^ and the native structure of T8 is the positioning of this acetyl group. We then purified the non-toxic T8^Y140F^ mutant protein, which can be produced in large quantities in the absence of its antitoxin A8, and solved its crystal structure to 1.7 Å resolution (Table 1; Figure S2A). The asymmetric unit of the crystals contains a dimer of T8 with an extensive dimeric interface burying a total of 4,739 Å^2^ of surface area (Figure 2B). The T8 monomer consists of an acetyltransferase fold, and an Ac-CoA molecule is clearly visible in the electron density of the active site (Figures 2B and S2B). Compared to that of other acetyltransferases, the structure contains two unique features: an extension to the α3 helix and longer, curved β2 and β3 strands with an insertion of short 3_10_- and α helices (η1 and α2) between them (Figure 2C). The dimer interface is also unique and critically depends on both the α3 and β2-β3 extensions. Hydrophobic sidechains from these structural components and from the α1-β1 loop are buried to stabilize the dimer in conjunction with several hydrogen bonds (Figures 2B and S3). Together, these observations suggest that T8 represents a unique class of acetyltransferases.

### T8 Acetylates tRNA in a Cell-Free Expression Assay

**Figure 3:**
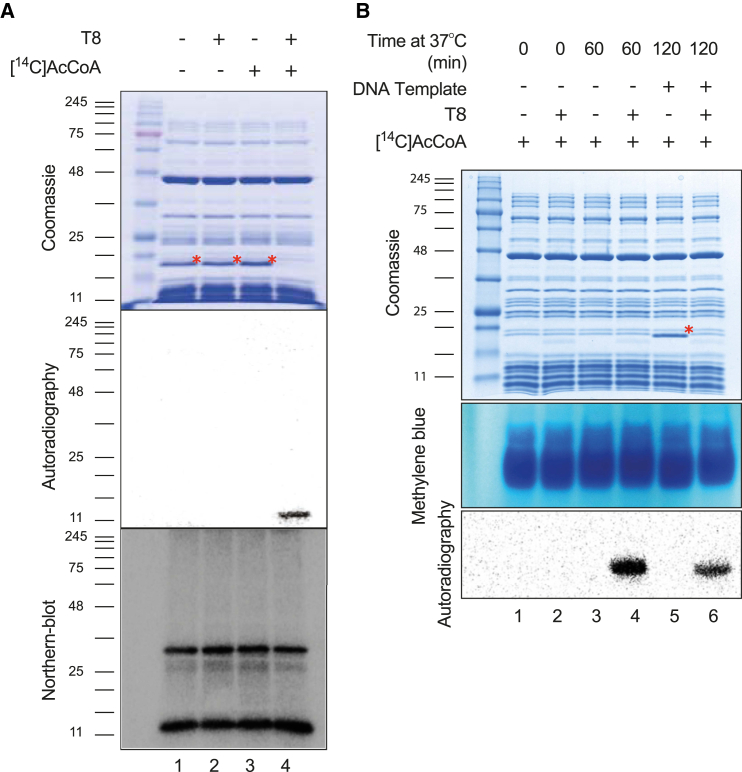
TacT Acetylates tRNA in Cell-Free Expression Assay (A) Cell-free expression assays leading to the production of the control protein DHFR (red asterisk) from template DNA without T8 (lanes 1 and 3) or with T8 (lanes 2 and 4) added from the onset of the assay. [^14^C]Ac-CoA was added to lanes 3 and 4. All samples were analyzed by SDS-PAGE. Production of DHFR was revealed by Coomassie staining (top panel) and acetylation by autoradiography (middle panel). tRNA molecules were detected by northern blotting using a radiolabeled probe specific of tRNA^Ala^ (lower panel). (B) Acetylation of tRNA molecules during cell-free expression was tracked by addition of [^14^C]Ac-CoA in all samples. Samples were only supplemented with template DNA to fire expression after 60 min of incubation (lanes 5 and 6) of all components of transcription and translation machineries without T8 (lanes 1, 3, and 5) or with T8 (lanes 2, 4, and 6) added to the samples from the onset of the assay. After 0, 60, or 120 min of incubation, production of DHFR was detected by SDS-PAGE followed by Coomassie staining (top panel). Concomitantly, extracted tRNA molecules were separated on acid-urea polyacrylamide gel and revealed by methylene blue staining (middle panel) followed by autoradiography (lower panel). Red asterisks mark the position of the DHFR in all panels containing Coomassie-stained gels (see also Figure S4).

To elucidate how T8-mediated acetylation interferes with translation, we purified the wild-type toxin after co-expression with its cognate antitoxin to prevent impairment of growth of the recombinant *E. coli* strain. The toxin formed a stable complex with its antitoxin (Figure S4A). This was dissociated through denaturation on a nickel column followed by toxin refolding. We investigated the effect of purified T8 in a cell-free expression assay using production of dihydrofolate reductase (DHFR) as a readout of successful translation. T8 completely inhibited production of DHFR, but only when the toxin was supplemented with [^14^C]Ac-CoA (Figure 3A). This was accompanied by acetylation of a low molecular weight species, as evidenced by autoradiography (Figure 3A). In an attempt to identify the acetylated molecules, peptides were recovered from the corresponding region of the Coomassie-stained SDS-PAGE gel and analyzed by nano-HPLC electrospray ionization multistage tandem mass spectrometry (nLC-ESI-MS/MS). All peptides were from ribosomal proteins, but no differential acetylation was detected following incubation with T8 (data not shown). However, tRNA molecules, specifically probed by northern blotting, also migrate with very low apparent molecular mass during SDS-PAGE (Figure 3A). We analyzed tRNA molecules recovered from cell-free assays directly by northern blotting after acid-urea PAGE to test for the accumulation of abortive peptidyl-tRNA that could result from elongation arrest. If so, an incremental increase in the molecular mass of distinct tRNA molecules should be detected, where the size of the shift for each specific tRNA would reflect the length of the peptide chain attached to it (tripeptidyl-tRNA^Ser^, tetrapeptidyl-tRNA^Leu^, etc.). The migration pattern of several distinct tRNA molecules indicated that the bulk of tRNA molecules were charged when translation was inhibited, but no peptidyl-tRNA accumulation was observed (Figure S4B). Analysis of cell-free translation assay samples treated with [^14^C]Ac-CoA and T8 revealed significant acetylation of tRNA (Figure 3B). This acetylation occurred when no DNA template was added to the assay, indicating that neither active transcription nor translation was required (Figure 3B). Collectively, these results show that inhibition of protein synthesis in vitro by T8 is accompanied by acetylation of tRNA molecules; therefore, the toxin was renamed TacT (tRNA-acetylating toxin).

### Dimers of TacT Exhibit a Positively Charged Surface Essential for Toxicity

**Figure 4:**
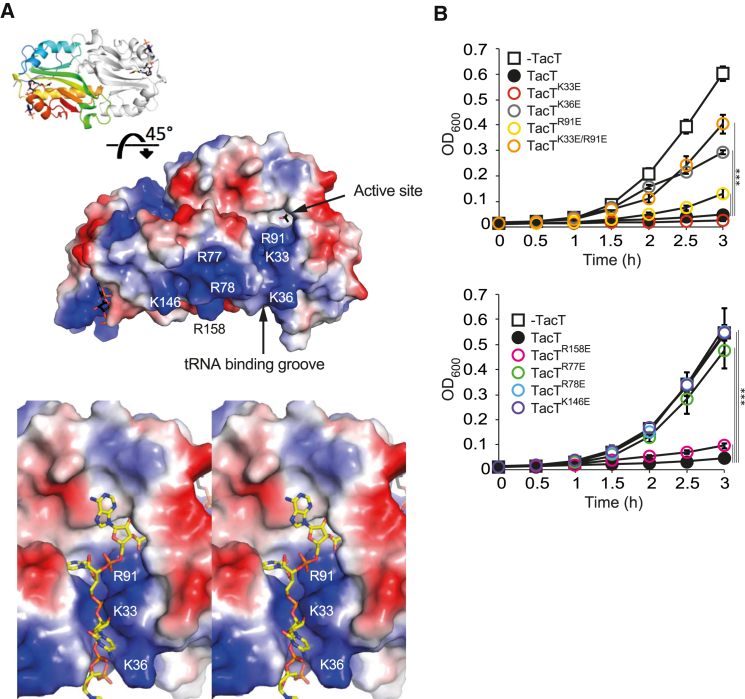
Positive Patches at the Surface of TacT Dimers Are Essential for Toxicity (A) Electrostatic potential of the surface of the TacT dimer showing positive potential in blue, negative in red, and residues mentioned in the text labeled. Top panel shows the overall dimer structure (a 45° rotation about the x axis from Figure 2B). Bottom panel shows a walleye stereo image with a charged tRNA acceptor stem (yellow sticks) modeled in the active site. (B) Effect of amino acid substitutions on TacT toxicity. Growth curves of *S*. Typhimurium 12023 Δ*tacAT* expressing from pNDM220, the wild-type toxin (+TacT), or point mutant toxins, or carrying the empty vector (−TacT). All cultures were supplemented with IPTG in fresh rich medium during lag phase. Kinetics illustrate the effect of toxins bearing substitutions in the positively charged groove (top panel: R91E, K33E, K36E, R91E/K33E), or in the second positive patch of the second TacT monomer (lower panel: R77E, R78E, R158E, K146E). Data represent the mean ± SEM (n ≥ 3) and were analyzed using a Student’s t test (^∗∗∗^p < 0.005) (see also Figure S5).

Examination of the surface electrostatic potential of the TacT dimer revealed a positively charged groove leading from the active site of one monomer to an extra patch of positive charge around the α2 region of the second molecule of the dimer (Figure 4A). The dimensions of the patches are compatible with the binding of a tRNA molecule. We modeled the coupled acceptor stem of the phosphodiester backbone lying in the groove with phosphate groups interacting with the sidechains of Arg-91, Lys-33, and Lys-36 (Figure 4A) and the positive surface of the α2 region positioned to interact with phosphates from the remainder of the tRNA molecule, with sidechains of Arg-77, Arg-78, Lys-146, and Arg-158 involved. This would necessitate TacT operating as a dimer. Consistently, size-exclusion chromatography with multi-angle laser light scattering (SEC-MALLS) analysis indicated that TacT is dimeric in solution (Figure S5). In this model, the target amine group of an aminoacyl-tRNA (charged tRNA) molecule is in close proximity to the acetyl moiety of Ac-CoA.

To test our model, we analyzed the effect of amino acid substitutions, both in the groove and on the surface close to the α2 region, on TacT toxicity. Whereas R91E, R158E, and K33E had little (although significant) to no effect on TacT toxicity, the single amino acid substitutions K36E, R77E, R78E, and K146E, or double substitution R91E/K33E, abolished TacT toxicity in *Salmonella* completely (Figure 4B). These results provide experimental support for a model of tRNA binding to TacT by electrostatic interactions.

### TacT Acetylates Aminoacyl-tRNAs in *Salmonella*

**Figure 5:**
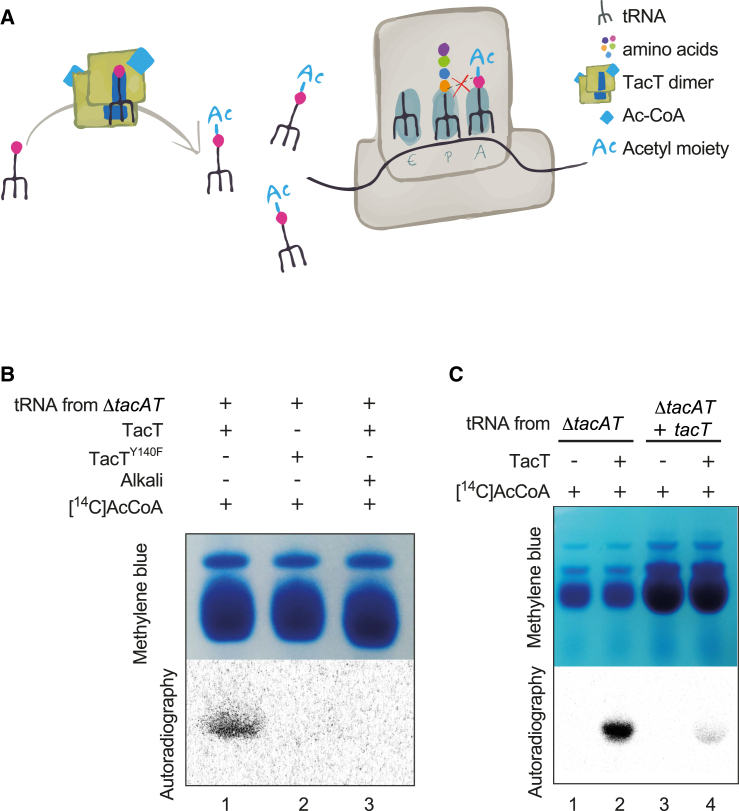
TacT Acetylates the Amino Acid Charged onto *Salmonella* tRNAs (A) Proposed model of action of TacT. TacT inhibits translation by acetylation of the primary amine group of amino acids charged onto tRNA molecules. The negatively charged tRNA molecule binds to TacT through its positive surface residues such that the primary amine group of the amino acid carried by the tRNA sits in the active site of the acetylase. Upon acetylation, the alpha amine group engaged with the acetyl moiety is blocked and cannot react for peptide bond formation with the carboxyl group of the amino acid loaded on the tRNA in the ribosomal P site. (B) tRNA molecules were extracted from *S*. Typhimurium 12023 Δ*tacAT* and preserved (lanes 1 and 2) or alkali treated in vitro (lane 3) before subsequent incubation with purified TacT (lanes 1 and 3) or purified TacT^Y140F^ (lane 2). (C) Acetylation of tRNA molecules extracted from *Salmonella* by purified TacT. tRNAs were extracted from *S*. Typhimurium 12023 Δ*tacAT* (lanes 1 and 2) or *S*. Typhimurium 12023 Δ*tacAT* carrying pNDM220::*t8* (+TacT) (lanes 3 and 4), then incubated in vitro with purified TacT. All samples were supplemented with [^14^C]Ac-CoA. Treated tRNA molecules were separated on acid-urea polyacrylamide gel and revealed by methylene blue staining (top panels), and TacT-dependent acetylation was revealed by autoradiography (lower panels).

Our model of the toxin dimer interacting with tRNA suggests that TacT acetylates the primary amine group of the amino acid on the charged nucleic acid molecules (Figure 5A). At each cycle of normal translation elongation, a new charged tRNA molecule binds to the ribosomal A site while the nascent peptidic chain is bound to the tRNA located in the P site. The amine group of the A site amino acid and the carboxyl group of the most recently incorporated amino acid in the polypeptide chain react to form a peptide bond. If acetylated, the amine group of the A site amino acid would be unavailable to the carboxyl group and protein synthesis would be blocked (Figure 5A). This is the basis for translation arrest by chemical acetylation of aminoacyl-tRNA (Cuzin et al., 1967, van der Laken et al., 1980).

Acetylation is difficult to track in vivo; however, purified TacT, but not TacT^Y140F^, acetylated free tRNA molecules extracted from Δ*tacAT Salmonella* (Figure 5B). Purified TacT did not acetylate tRNAs that had been previously uncharged by alkaline treatment (Figure 5B), indicating that TacT acetylates the amino acid on charged tRNA molecules extracted from *Salmonella*. Although overall amounts of tRNA increased considerably upon overproduction of TacT (Figure 5C), there was a significant overall reduction in the acetylation of tRNAs extracted from *tacT*-overexpressing bacteria (Figure 5C, lane 4), compared to that observed on tRNAs extracted from Δ*tacAT Salmonella* (Figure 5C, lane 2). This shows that tRNAs exposed to TacT in vivo were much less susceptible to subsequent activity of TacT in vitro, suggesting that the acetylation had already occurred in the bacteria. Together, these results indicate that TacT acetylates the amino acid on charged tRNA molecules in *Salmonella*.

### Detoxification of TacT-Dependent Toxicity

The entry of bacteria into growth arrest is readily explained through the activity of toxins targeting vital cellular processes such as translation, but the mechanism(s) of detoxification that allows persisters to resume growth is unclear. It has been proposed that growth resumption could occur upon replenishment of the pool of antitoxin, thereby neutralizing the toxin. However, such an event is difficult to reconcile with a block in translation (Cataudella et al., 2012), and it is possible that other mechanisms contribute to neutralization of toxin activity.

**Figure 6:**
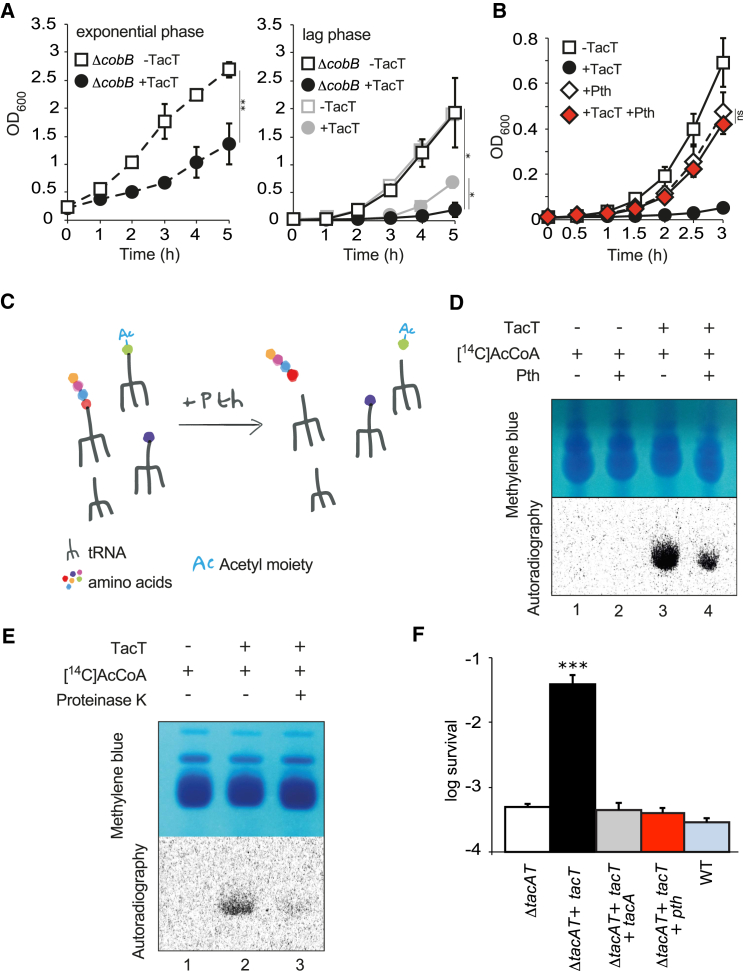
Pth Counteracts TacT Toxicity and TacT-Dependent Persister Formation (A) Growth curves of *S*. Typhimurium 12023 Δ*ta8*Δ*cobB* carrying pNDM220 (Δ*cobB* − TacT) or pNDM220::*t8* (Δ*cobB* +TacT) in fresh rich medium. Expression of *tacT* was induced upon addition of IPTG during mid-exponential (left panel) or lag (right panel) phase. Growth curves from Figure 1A, right panel, were reported in gray for comparison purposes. Data represent the mean ± SEM (n ≥ 3) and were analyzed using a Student’s t test (^∗^p < 0.05; ^∗∗^p < 0.01). (B) Growth curves of *S*. Typhimurium 12023 Δ*tacAT* carrying pNDM220 (−TacT), pNDM220::*t8* (+TacT), pBAD33::*pth* (+Pth), or pNDM220::*t8* and pBAD33::*pth* (+TacT +Pth). All cultures were supplemented with arabinose and IPTG in fresh rich medium during lag phase. Data represent the mean ± SEM (n ≥ 3) and were analyzed using a Student’s t test (ns, non-significant). (C) Pth hydrolyses the ester bond between the nascent peptide and tRNA upon release of free peptidyl-tRNA molecules. Pth does not deacylate charged tRNA, but recognizes and cleaves acetylated aminoacyl-tRNA molecules as dipeptidyl-tRNA. (D) Exposure of tRNA molecules acetylated by TacT to Pth treatment in vitro. Cell-free expression assays leading to the production of the control protein DHFR were supplemented with TacT (lanes 3 and 4) from the onset of the assay, then tRNA molecules were extracted and TacT-dependent acetylation was assessed by autoradiography, before (lane 3) or after (lane 4) samples were treated with Pth (lower panel). (E) Exposure of tRNA molecules acetylated by TacT to Proteinase K treatment in vitro. tRNA molecules were extracted from *S*. Typhimurium 12023 Δ*tacAT* and incubated in vitro with purified TacT (lanes 2 and 3). Proteinase K was subsequently added to one sample (lane 3). (D and E) All samples were supplemented with [^14^C]Ac-CoA. Treated tRNA molecules were separated on acid-urea polyacrylamide gel and revealed by methylene blue staining (top panels), and TacT-dependent acetylation was revealed by autoradiography (lower panels). (F) Proportion of bacteria surviving 4 hr exposure to bactericidal concentrations of cefotaxime in cultures of *S*. Typhimurium 12023 Δ*tacAT*, Δ*tacAT* pNDM220::*tacT*, pNDM220::*tacT* and pBAD33::*tacA*, pNDM220::*tacT* and pBAD33::*pth*, or wild-type. Arabinose and IPTG were added to all cultures in fresh medium during lag phase, then antibiotic treatment started 1 hr later. Data represent the mean ± SEM (n ≥ 3) and were analyzed using a Student’s t test (ns, non-significant; ^∗∗∗^p < 0.001) (see also Figures S6 and S7).

Acetylation can either occur co- or post-translationally. Co-translational acetylation usually takes place on the alpha amine group of the N-terminal residue and is a permanent modification, whereas post-translational acetylation occurs on the epsilon amine group of lysine residues and is reversible. We determined if the effect of TacT is reversible through the activity of an endogenous deacetylase that could detoxify TacT target(s). *Salmonella* only has one characterized non-specific deacetylase, CobB (Starai and Escalante-Semerena, 2004). Whereas overexpression of *tacT* did not alter growth during exponential phase (Figure 1A), deletion of *cobB* abolished the dependency of TacT toxicity on bacterial growth phase and led to prolonged activity of TacT on *S.* Typhimurium lag phase (Figure 6A); however, no direct counteraction of toxicity was observed upon *cobB* overexpression. After 5 hr of induction, TacT appeared to lose its inhibitory effect on bacterial growth even in the absence of CobB (Figure 6A), and after addition of more inducer (data not shown). These results suggest that the endogenous deacetylase CobB contributes to detoxifying the cells, but that other counteracting factors may exist.

It has been shown previously that toxin-induced bacterial growth arrest can be rescued by overexpression of the target (Germain et al., 2013). We used a similar approach and screened the ASKA collection (Kitagawa et al., 2005) of high-copy-number plasmids containing most *Escherichia coli* open reading frames (ORFs) and selected for constructs that suppressed TacT toxicity. This was possible as TacT induces an extension of lag phase that had noticeable effects on the timing of colony recovery on plates. As TacT targets tRNAs (whose coding regions are not represented in the library), we hypothesized that the suppressor screen could lead to the identification of detoxifying mechanisms. The pCA24N::*pth* plasmid was specifically selected for in the screen and suppressed TacT-induced toxicity in *E. coli.*

Peptidyl-tRNA hydrolase (Pth) is an essential esterase of all bacterial species that recycles free peptidyl-tRNA molecules released during premature termination of translation (Sharma et al., 2014). A variety of factors frequently perturb translation, leading to ribosome stalling. Unless rescued by *trans*-translation, this leads to the release of toxic peptidyl-tRNA molecules (Giudice and Gillet, 2013). In such cases, Pth hydrolyzes the ester bond between the carboxy-terminal end of the peptide and the 3′ hydroxyl of tRNA, thus preventing a shortage of available tRNA and amino acids that would otherwise lead to a halt both in translation and bacterial growth (Giudice and Gillet, 2013, Sharma et al., 2014). Overexpression of *S.* Typhimurium *pth* had a small effect on bacterial growth rate. However, it counteracted the growth inhibitory effect of T8 (Figure 6B), but not that of the unrelated ParE toxin (Figure S6A).

To test if Pth is an additional target of TacT, His-tagged Pth was purified from *S.* Typhimurium with or without overexpression of *tacT* and samples were analyzed by nLC-ESI-MS/MS. We did not detect any acetylation of Pth (data not shown). We then tested if peptidyl-tRNA molecules accumulated upon TacT-induced growth arrest of *Salmonella*. As a positive control, we designed a *Salmonella* strain producing a thermosensitive form of Pth (Cruz-Vera et al., 2000). Upon shifting to a non-permissive temperature, we detected an accumulation of nascent peptides of different lengths linked to tRNA, as evidenced by a smear of tRNA molecules on a methylene-blue-stained acid-urea gel (Janssen et al., 2012) (Figure S6B). As previously observed (Figure 5C), overall amounts of tRNA increased considerably in *tacT*-overexpressing bacteria compared to a strain producing no TacT, but no peptidyl-tRNA accumulation was observed, suggesting that Pth activity was not impaired (Figure S6B). The potential effect of TacT on Pth hydrolase activity was tested further using in vitro activity assays. We purified recombinant *Salmonella* Pth and confirmed that the enzyme cleaved extracted peptidyl-tRNA substrate efficiently (Figure S6C). Exposure to TacT did not impair hydrolysis (Figure S6C). Moreover, in agreement with our observations in vivo, we did not detect any acetylation of purified Pth by providing TacT with [^14^C]Ac-CoA (data not shown). Altogether, these results show that Pth is not targeted directly by TacT but rather counteracts its toxicity.

It has been reported previously that whereas Pth does not deacylate charged tRNA, it hydrolyses peptidyl-tRNA and acetylated aminoacyl-tRNA molecules that are recognized as dipeptidyl-tRNA (Cuzin et al., 1967, Ito et al., 2012; Figure 6C). We precipitated free tRNA molecules acetylated by TacT during cell-free expression assays (Figure 6D) and incubated them with purified Pth in vitro. A strong and reproducible decrease in the radioactive acetylation signal was detected (Figure 6D), showing that Pth detoxified the acetylated charged tRNAs. A similar effect was apparent upon treatment with Proteinase K (Figure S6D), whose activity on tRNA molecules is very similar to Pth (Vidales et al., 1979; Figure S6E). tRNA molecules extracted from Δ*tacAT Salmonella* and subsequently acetylated by TacT in vitro (Figure 6E) were incubated with Proteinase K. Again, a strong decrease in the radioactive acetylation signal was detected (Figure 6E). Hence, we conclude that two enzymes that usually target and recycle N-blocked charged tRNAs resolve the TacT-driven acetylation of tRNAs, indicating that TacT modifies the primary amine group of the amino acid on charged tRNA molecules.

### Pth Counteracts TacT-Dependent Persister Formation

TA modules stimulate formation of antibiotic-tolerant persister bacteria (Helaine et al., 2014, Maisonneuve et al., 2011) through growth arrest induced by the activity of toxins (Maisonneuve et al., 2013). Accordingly, overexpression of *tacT* led to an increase in the proportion of *Salmonella* surviving exposure to bactericidal concentrations of three different classes of antibiotic during growth in vitro without having affected the minimum inhibitory concentration (MIC) (Figures 6F and S7). This corresponded to an increase in persister proportions, as illustrated by the biphasic killing curves obtained (Figure S7). Accordingly to what we previously reported, there was no decrease in in vitro persister formation in a *tacAT* deletion mutant compared to a wild-type strain as endogenous expression of *tacT* is specifically activated during macrophage uptake (Helaine et al., 2014). The increase in persister formation induced by overexpression of *tacT* was fully counteracted by overexpression of *tacA* (tRNA-acetylating antitoxin). Since we showed that Pth counteracts TacT-dependent toxicity by recycling acetylated charged tRNAs, we investigated its effect on persister formation. TacT-dependent persister formation was abolished by overexpression of *pth* (Figure 6F). This suggests that the balance between TacT, its cognate antitoxin TacA, and Pth modulates growth arrest and persister formation of *Salmonella*.

## Discussion

**Figure 7:**
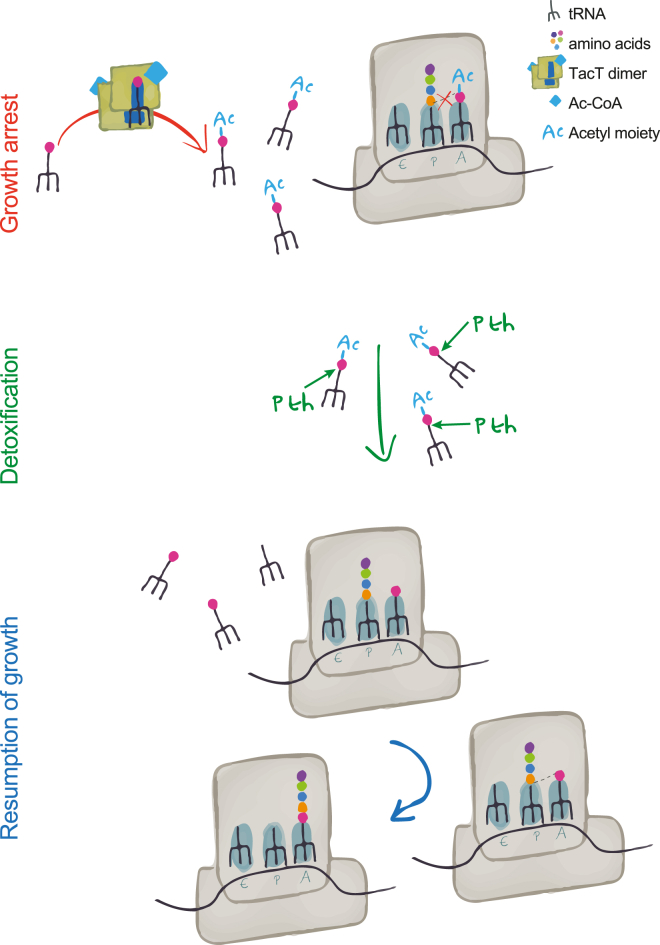
Model of TacT-Dependent Persister Formation and Resumption of Growth TacT, an acetyltransferase, inhibits translation by modification of the primary amine group of amino acids charged onto tRNA molecules. Upon acetylation (red arrow), the alpha amine group is blocked and cannot form a peptide bond with the nascent peptide, and bacteria stop growing. TacT activity on translation is counteracted by the action of Pth, which detoxifies the corrupted tRNAs (green arrow). When the effect of Pth is greater than that of TacT and detoxification is complete, translation resumes (blue arrow) and persisters regrow.

In this paper we show that TacT, which contributes to the formation of intracellular *Salmonella* persisters (Helaine et al., 2014), functions as the toxin component of a TA module. It arrests bacterial growth by blocking translation through acetylation of the primary amine group of charged tRNA (Figure 7).

Many class II TA toxins inhibit bacterial growth by disrupting translation. For example, some endoribonucleases cleave mRNA when free or associated with ribosomes (Yamaguchi and Inouye, 2011) and *E. coli* Doc phosphorylates EF-Tu (Castro-Roa et al., 2013). Several toxins target tRNA. These include *Salmonella* VapC, which cleaves the initiator tRNA^fMet^ (Winther and Gerdes, 2011), and *M. tuberculosis* MazF-mt9, which hydrolyses a subset of tRNAs (Schifano et al., 2016). *E. coli* HipA interferes with acylation of tRNA^Glu^ by phosphorylating glutamyl-tRNA synthetase, thereby inhibiting its aminoacylation activity (Germain et al., 2013, Kaspy et al., 2013).

Acetylation has emerged as a widespread post-translational modification in bacteria, altering hundreds of peptides in *E. coli*, *S.* Typhimurium, and *M. tuberculosis*, with targets involved in processes as diverse as metabolism, transcription regulation, or translation (Zhang et al., 2009, Wang et al., 2010, Xie et al., 2015). tRNAs can also be acetylated on nucleosides of the anticodon at the wobble position, thereby increasing the accuracy of their decoding activity (Kawai et al., 1989). Here we show that bacteria can acetylate the amino acid on charged tRNA molecules through the activity of TacT, an atypical acetyltransferase that displays unique structural features including two positively charged patches at its surface that are essential for toxicity. Our work suggests that upon activation of TacT, the negatively charged tRNA molecule docks onto it through its positive surface residues such that the primary amine group of the amino acid carried by the tRNA sits in the active site of the acetylase. TacT forms a dimer, suggesting that it might acetylate two tRNAs simultaneously; however, we hypothesize that the main function of the dimer is to constitute a complete binding site for the substrate. We modeled the coupled acceptor stem of the tRNA lying in the positively charged groove of one monomer with its phosphate groups interacting with sidechains of positively charged amino acids, and the remainder of the tRNA molecule interacting with the second positive surface of the second monomer of the dimer. Dimerization of Gcn5-related acetyltransferase with cooperativity of the two subunits has been reported previously in the case of the *Enterococcus faecium* AAC(6’)-Ii, which acetylates aminoglycosides (Draker et al., 2003). Residues of TacT that are involved in dimerization are conserved in the *Salmonella* toxins T6 and T9, and models of their structures based on that of TacT reveal positively charged patches on their surface. However, the amino acid sequences of the three GNAT toxins are dissimilar enough to justify a full investigation into the structure/function of T6 and T9.

TacT-catalyzed acetylation occurs on the free form of tRNAs. We propose that following acetylation, the alpha amine group blocked by the acetyl moiety cannot react to form a peptide bond with the carboxyl group of the amino acid loaded on the tRNA in the ribosomal P site, thereby aborting translation (Figure 7). Although our assays do not ascertain whether acetylated aminoacyl-tRNA still binds the A site of ribosomes, it was shown more than 30 years ago that chemical acetylation of *E. coli* tRNAs did not prevent in vitro association with purified EF-Tu and ribosomes. However, GTP hydrolysis by EF-Tu was compromised, thereby preventing the aminoacyl-tRNA from fully entering the ribosomal A site (Campuzano and Modolell, 1981). It is therefore possible that the block in translation induced by TacT happens before the more obvious defect of peptide bond formation and that EF-Tu remains locked on acetylated aminoacyl-tRNA after association with the ribosome. If this were the case, it might explain the increase in the overall amounts of tRNA we observed upon overproduction of TacT, as EF-Tu could protect tRNA molecules from degradation. Production of excess EF-Tu did not counteract TacT-induced toxicity (data not shown).

It remains to be determined if all aminoacyl-tRNA species can be acetylated by TacT in *Salmonella*. HipA and VapC, the other enteric toxins targeting tRNA, affect, directly or indirectly, only one specific tRNA but nevertheless impose a strict control on bacterial translation (Germain et al., 2013, Winther and Gerdes, 2011). In our model, the target amine group of a charged tRNA molecule is in close proximity to the acetyl moiety of Ac-CoA and the active site of TacT. Whereas our model (Figure 4A) is of an alanine-charged tRNA, there is sufficient space in the active site to accommodate larger side chains without taking account of the likely local structural plasticity upon substrate binding.

The mechanism used by TacT to block translation might be shared by many other bacterial species and represent a paradigm of growth control, regardless of whether the acetyltransferase is part of an identified TA module. GNAT toxins are present in many pathogenic species from proteobacteria (i.e., *Salmonella*, *Shigella*, or *Vibrio*) to actinobacteria (i.e., mycobacteria), and even in archae. Although a few have been reported to have an impact on bacterial growth (Iqbal et al., 2015, Lobato-Márquez et al., 2015), to date no acetylated target has been reported, and the mechanism of action of TacT could be widespread throughout phyla.

We have reported previously that TacAT is specifically activated upon entry of *Salmonella* in macrophages contributing to persister formation. We showed intracellular persister formation to require Lon, an ATP-dependent protease and an active stringent response (Helaine et al., 2014). This was in agreement with the model proposed for *E. coli* persister formation where activation of the stringent response and synthesis of (p)ppGpp by starvation leads to accumulation of polyphosphate and Lon-mediated class II antitoxin degradation (Maisonneuve et al., 2013). Class II antitoxins interact with and neutralize their cognate toxin (Yamaguchi and Inouye, 2011), providing both immunity from the toxin and a means to resume bacterial growth from the toxin-induced arrest. However, whereas the entry of bacteria into growth arrest is readily explained through the activity of toxins, the mechanism by which persisters resume growth is more mysterious. Growth resumption could occur upon replenishment of the pool of antitoxin; however, such an event is difficult to reconcile with the toxin-induced translation arrest (Cataudella et al., 2012). We considered that other mechanisms might contribute to neutralization of toxin activity, particularly as the modification seemed conservative of the integrity of the tRNA as opposed to the activity of nuclease toxins, such as VapC, for example. We uncovered that an additional protein, Pth, has a detoxifying activity and contributes to growth resumption from TacT-induced growth arrest.

Pth allows recycling of acetylated tRNA molecules and thereby detoxifies the cell. Following acetylation of charged tRNAs by TacT, Pth activity will release uncharged tRNAs in the cell that can be charged again by aminoacyl-tRNA synthesases. HipA leads to the increase in uncharged tRNA^Glu^, thereby driving stringent response activation and persister formation in *E. coli* (Germain et al., 2015). We have no evidence to date that the activity of TacT triggers the stringent response and activates more TA module. Moreover, we showed that Pth counteracts TacT-induced persister formation rather than amplifying it, and the balance between Pth and TacT is critical for persister formation (Figure 6F).

Similarly, deletion of *cobB*, the gene coding for the endogenous CobB sirtuin deacetylase, leads to increased toxicity of TacT (Figure 2D). One interesting possibility raised by this observation is that after TacT-induced arrest, CobB also contributes to detoxification of acetylated charged tRNAs. Sirtuins are reported to hydrolyse *N*^ε^-lysine acetylation of proteins. Whether acetylated aminoacyl-tRNAs are direct substrates to CobB or the deacetylase acts through another mechanism remains to be determined. Interestingly, it has been reported that *S.* Typhimurium produces two active isoforms of CobB of different lengths. The long isoform CobBL displays an N-terminal positively charged extension comprising many Arg residues. The authors proposed that, similarly to nonribosomal, Arg-rich peptides with comparable amino acid compositions, the N terminus of CobBL could bind nucleic acids (Tucker and Escalante-Semerena, 2010). This is consistent with our observations, and a direct role of CobB in detoxifying acetylated aminoacyl-tRNA merits further investigation. The mechanism behind the switch, from a high level of TacT activity to Pth (and possibly CobB) taking over, remains to be elucidated. However, as opposed to a model in which replenishment of the pool of antitoxin leads to toxin neutralization and exit from the persister state, detoxification by Pth and CobB would not necessarily require new translation to exit TacT-dependent growth arrest. Different half-lives of the three proteins could explain a premature loss of activity of TacT compared to CobB and Pth. However, the balance between essential cofactors (namely Ac-CoA and NAD^+^ for TacT and CobB, respectively) might also have an important role. Indeed, the enzymatic activity of CobB, like that of other NAD^+^-dependent sirtuins, probably relies on the cellular levels of NAD^+^. Therefore, if Ac-CoA levels become limiting in non-growing bacteria sooner than NAD+, the balance between TacT and CobB activity could tilt toward CobB. Understanding of the mechanisms that stimulate regrowth of persisters might have potential application in devising approaches to activate them artificially, to stimulate their growth and re-sensitize them to antibiotics.

### Final Perspective

TacT is a unique acetyltransferase that targets amino acids of charged tRNA. This provides *Salmonella* with an efficient way to block translation transiently and thereby halt growth upon activation of the TacAT module, notably during macrophage infection. *Salmonella* possesses several detoxifying enzymes to recycle the corrupted tRNAs and resume growth, a key condition for the arrested bacteria to be successful persisters. Promoting the activity of these detoxifying enzymes in TacT-induced *Salmonella* persisters would lead to resumption of growth and restore bacterial susceptibility to antibiotics.

## Experimental Procedures

### Bacterial Strains and Media

The *S*. Typhimurium strains used in this study were wild-type 12023s and its mutant derivatives (Table S1). The *E. coli* expression strain was BL21 (Table S1). All strains were grown at 37**°**C in fully aerated rich growth medium (Luria Bertani) or M9 minimal medium, supplemented when appropriate with 100 μg/mL ampicillin, 25 μg/mL chloramphenicol, 50 μg/mL kanamycin, 0.2% L-arabinose, and/or 0.5–1 mM IPTG to allow production of recombinant proteins.

### Rates of Protein, DNA, and RNA Synthesis

Rates of incorporation of Methionine-^35^S (protein synthesis), Thymidine-2-^14^C (DNA synthesis), or Uracil-2-^14^C (RNA synthesis) were measured over time after induction of toxin expression. More details are available in the Supplemental Experimental Procedures.

### Expression, Purification, and Crystal Structure Determination of Recombinant Toxin

Details are available in the Supplemental Experimental Procedures.

### Analysis of tRNA and Peptidyl-tRNAs by Gel Electrophoresis

Total RNA was extracted from *Salmonella* under acidic conditions to maintain the ester link between tRNA and amino acid/peptide. Procedures were taken from Janssen et al. (2012). Details are available in the Supplemental Experimental Procedures.

## Author Contributions

Purification of the toxin and crystallization experiments performed by M.P., C.T.W., and S.A.H. Purification of Pth performed by A.M. All other experiments performed by A.M.C., B.G., and S.H. A.M.C., B.G., S.A.H., and S.H. designed experiments and analyzed data. S.H. wrote the paper with input from all other authors.

## Figures and Tables

**Table 1:** T8^Y140F^ Data Collection and Refinement Statistics

	Native	Bromide SAD
**Data Collection**		

Space group	P2_1_	P2_1_
Cell dimensions: *a*, *b*, *c* (Å)	33.4, 85.7, 55.3	33.2, 85.2, 55.2
Cell dimensions: α, β, γ (°)	90, 93.1, 90	90, 92.1, 90
Resolution (Å)	55.2–1.70	85.2–2.06
(1.79–1.70)^a^	(2.11–2.06)
No. of unique reflections	32830 (4805)	19156 (1495)
R_pim_	0.058 (0.360)	0.085 (0.633)
*I* / σ*I*	8.6 (2.0)	20.4 (8.6)
Completeness (%)	96.2 (96.5)	99.9 (98.7)^b^
Redundancy	6.2 (6.0)	50.4 (47.9)^b^

**Refinement**		

Resolution (Å)	55.2–1.7	–
No. of reflections	30992	–
*R*_work_ / *R*_free_	0.172 / 0.225	–
No. of protein atoms	2557	–
No. of ligand/ion atoms	102	–
No. of water atoms	426	–
*B*-factors: protein	17.72	–
*B*-factors: ligand/ion	21.54	–
*B*-factors: water	29.24	–
RMSD: bond lengths (Å)	0.012	–
RMSD: bond angles (°)	1.542	–
Ramachandran plot: favored (%)	99.1	–
Ramachandran plot: allowed (%)	0.9	–
Ramachandran plot: outliers (%)	0	–

a Values in parentheses are for highest-resolution shell.

b Completeness and redundancy statistics from anomalous data.

## Accession Numbers

The coordinates for the structural model of TacT^Y140F^ have been deposited in the Protein Data Bank under ID code PDB: 5FVJ.

## Supplemental Information

Document S1. Supplemental Experimental Procedures, Figures S1–S7, and Table S1

Document S2. Article plus Supplemental Information
