# Supplementary material for: A Salmonella Toxin Promotes Persister Formation through Acetylation of tRNA
Source: Mol Cell. 2016 Jul 7;63(1):86–96. doi: 10.1016/j.molcel.2016.05.002 (PMC4942678; doi:10.1016/j.molcel.2016.05.002)
Supplement: Document S1. Supplemental Experimental Procedures, Figures S1–S7, and Table S1 [file mmc1.pdf]

Molecular Cell, Volume *63*

Supplemental Information

A *Salmonella* Toxin Promotes Persister Formation through Acetylation of tRNA

Angela M. Cheverton, Bridget Gollan, Michal Przydacz, Chi T. Wong, Anastasia Mylona, Stephen A. Hare, and Sophie Helaine

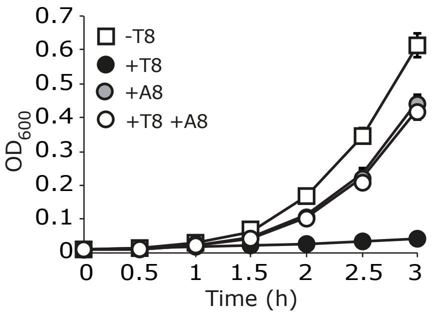

Figure S1. A GNAT-related toxin of *Salmonella* extends lag phase (related to Figure 1) Growth curves monitored by OD600 of *S*. Typhimurium 12023 Δ*ta8* carrying pNDM220 (-T8), pNDM220::*t8* (+T8), pBAD33::*a8* (+A8) or pNDM220::*t8* and pBAD33::*a8* (+T8+A8). All cultures were supplemented with arabinose and IPTG in fresh rich medium during lag phase. Data represent the mean ± SEM (n≥3).

A

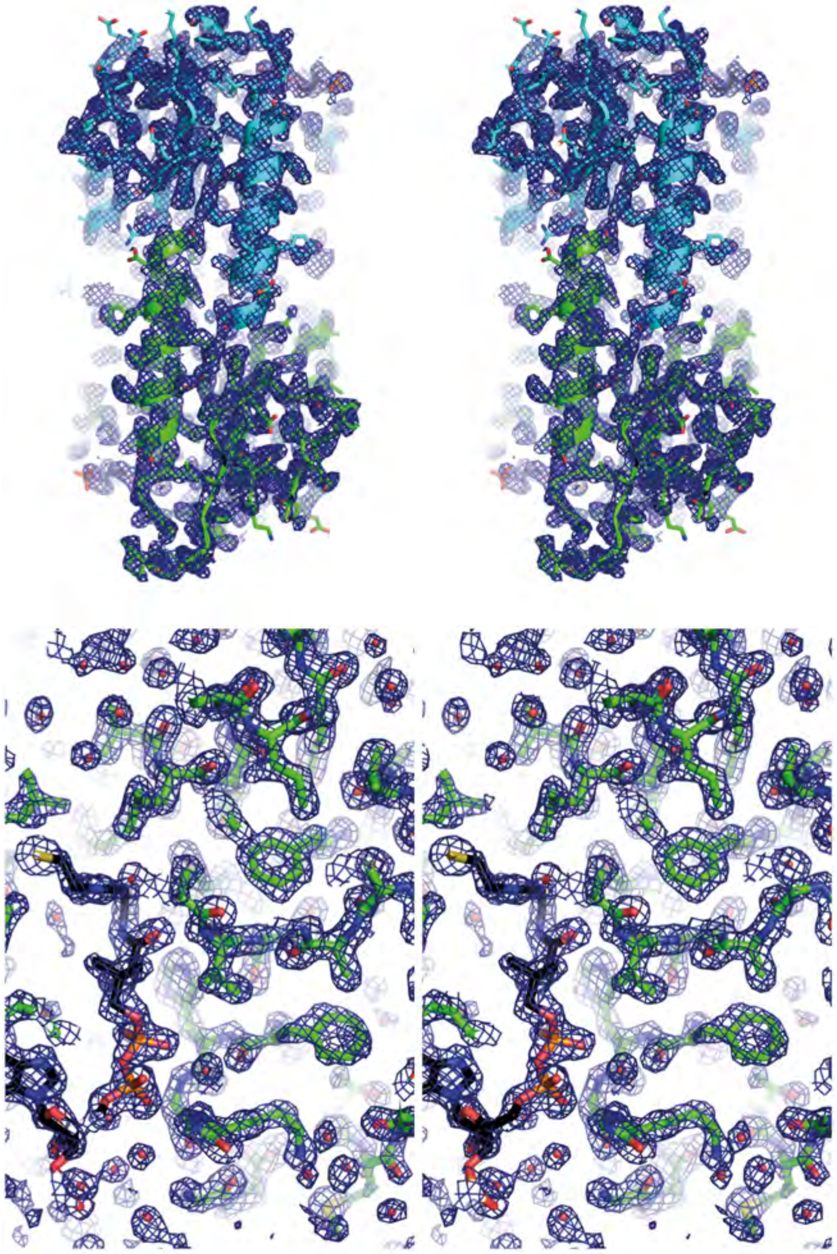

Density modification map

1.0 σ

Final map

1.2 σ

B

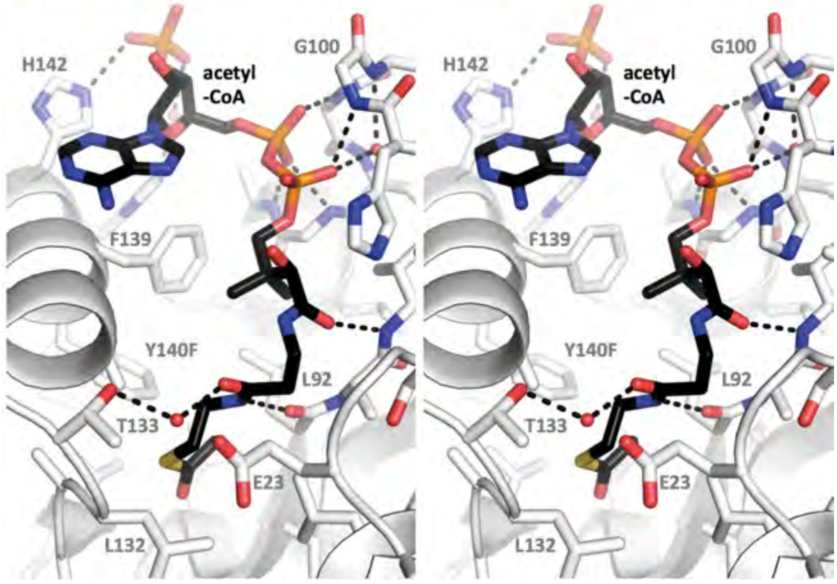

Figure S2. Walleye stereo view of T8^{Y140F} (related to Figure 2

(A) Walleye stereo view of the final T8^{Y140F} structure in the original density modified map contoured at 1σ (upper panel) and in the final $2F_O$-F_C map at 1.2σ. Protein chains are coloured cyan and green and Ac-CoA in black.

(B) A walleye stereo image of the T8^{Y140F} active site. Protein carbon atoms are light grey and those of Ac-CoA are black. Interacting residues are labelled and hydrogen bonds are shown as black dashed lines.

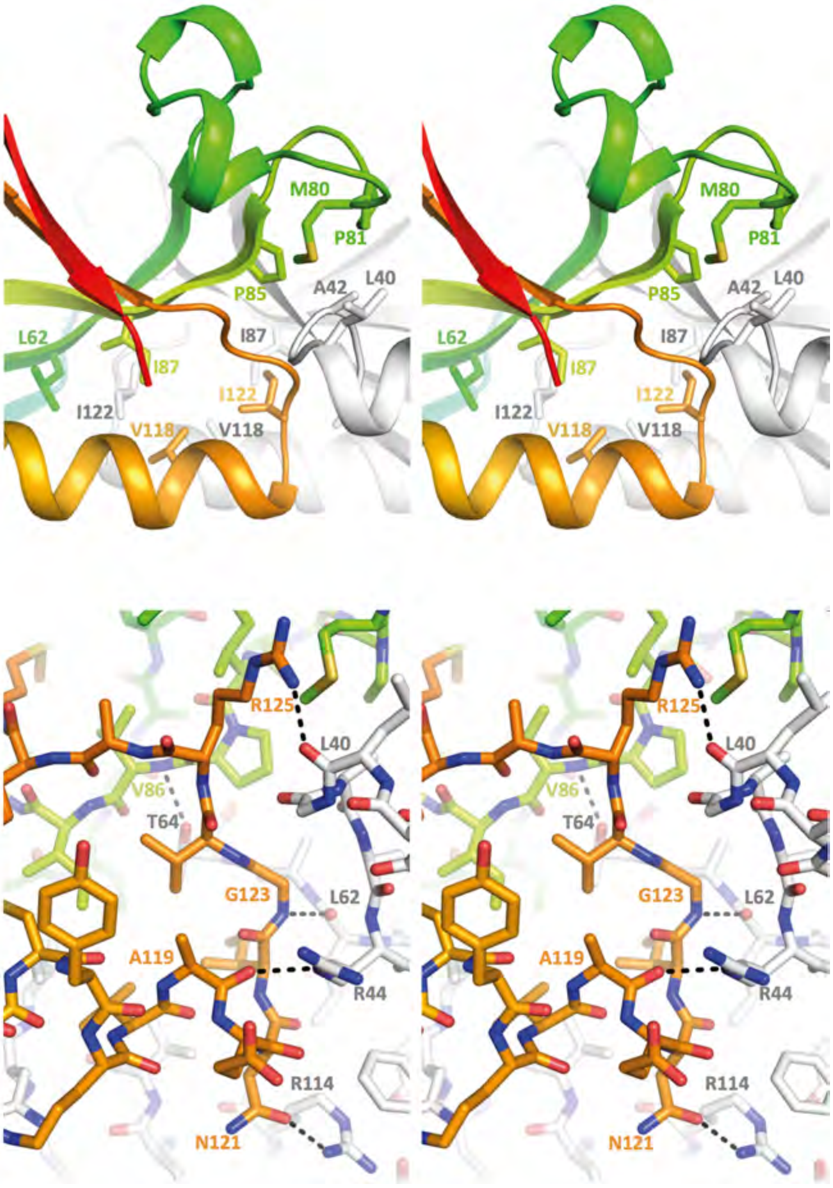

Figure S3. Details of the dimer interface (related to Figure 2)

Colours are conserved from Figure 2B (chain A rainbow, chain B grey) and the view conserved from Figure 2C. The upper panel highlights hydrophobic sidechains buried at the interface, whereas the lower panel shows hydrogen bonds stabilizing the interface.

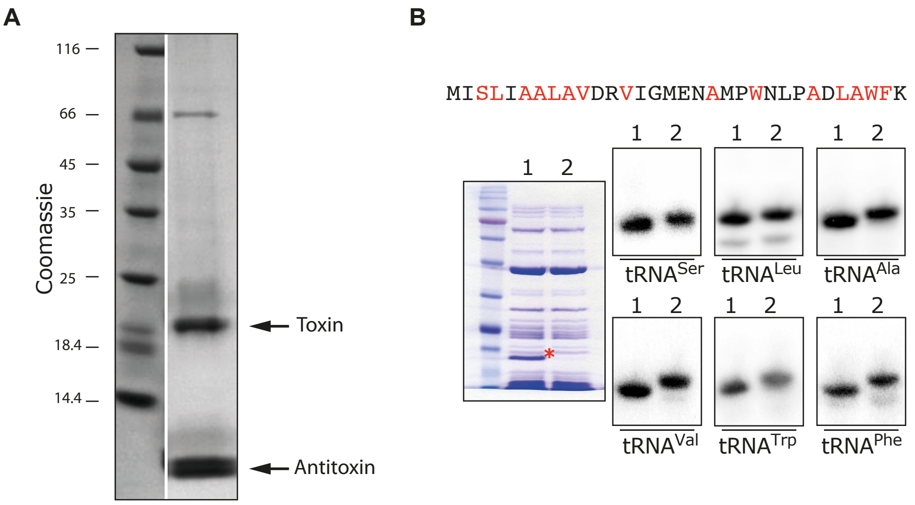

Figure S4. Purification of T8 in a complex with A8 and its effects on cell free expression (related to Figure 3)

(A) SDS-PAGE analysis of a collected fraction of T8 purification after affinity chromatography followed by size exclusion chromatography. The black arrows indicate the positions of toxin (T8) and the antitoxin (A8).

(B) Cell free expression assays leading to the production of the control protein DHFR whose first 32 residues are displayed at the top of the panel. All samples included T8. DHFR synthesis was allowed (sample 1) or inhibited by addition of Ac-CoA from the onset of the assay (sample 2). Samples were in part analysed by SDS-PAGE and Coomassie staining (left panel), or tRNA molecules recovered, separated on acid-urea polyacrylamide gel and revealed by hybridization of specific radiolabelled probes as indicated below each Northern blot (right panels).

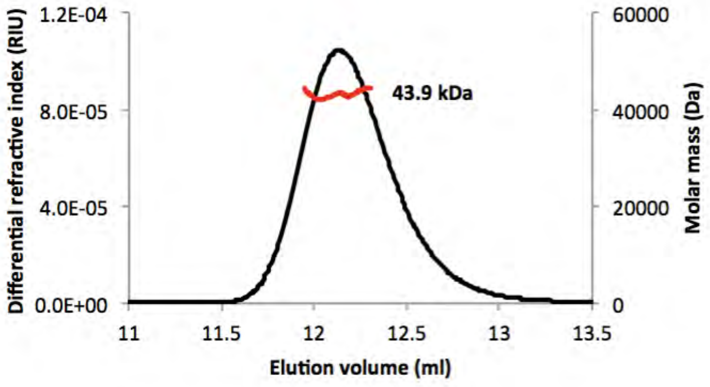

Figure S5. TacT forms a dimer in solution (related to Figure 4)
SEC-MALLS showing the elution profile of TacT^{Y140F} (black line) and predicted molecular weight (red line).

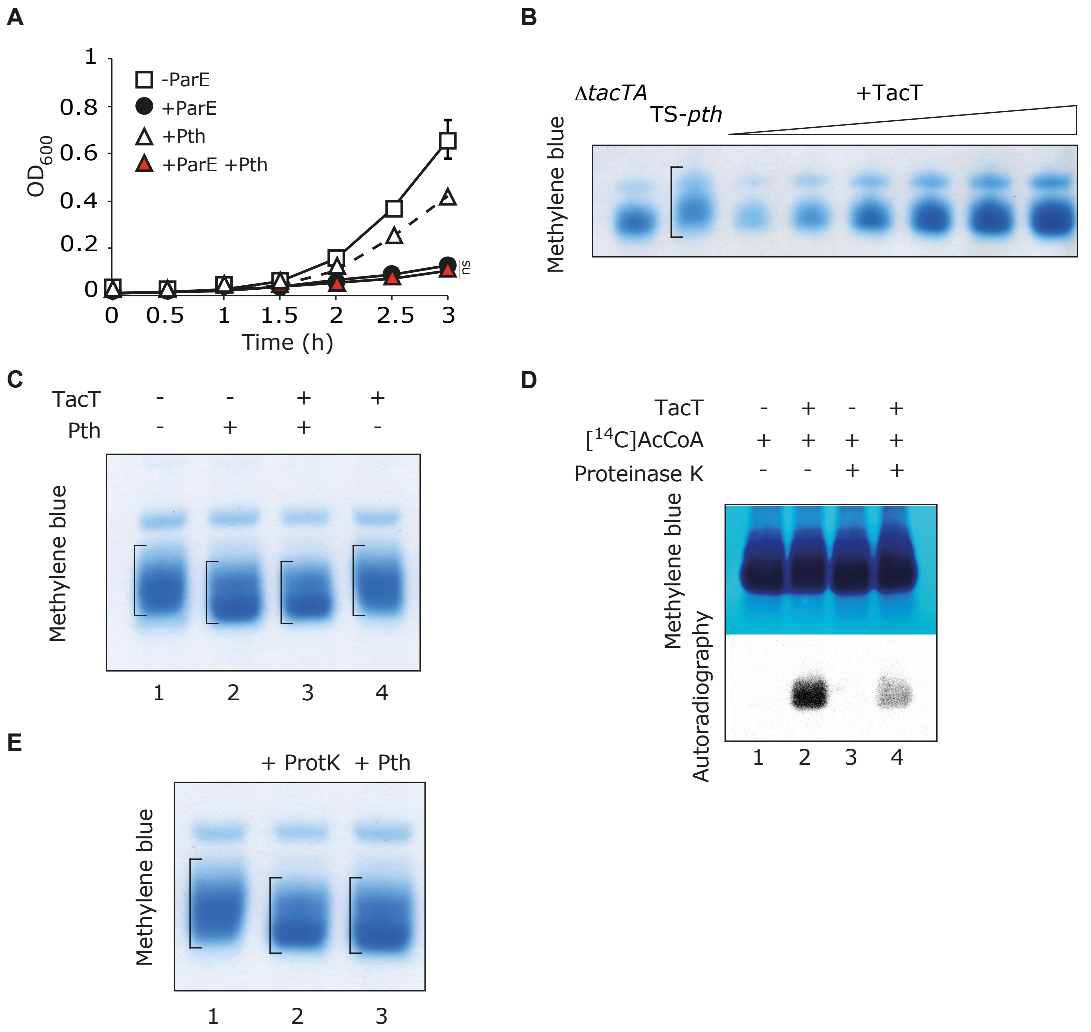

Figure S6. Pth is not a direct target of TacT (related to Figure 6)

(A) Growth curves of *S*. Typhimurium 12023 Δ*parDE* carrying pNDM220 (-ParE), pNDM220::*parE* (+ParE), pBAD33::*pth* (+Pth) or pNDM220::*parE* and pBAD33::*pth* (+ParE +Pth). All cultures were supplemented with arabinose and IPTG in fresh rich medium during lag phase. Data represent the mean ± SEM (n≥3) and were analysed using a Student's t test (ns, non significant).

(B) Analysis of peptidyl-tRNA accumulation. Total RNA was extracted from Δ*tacAT* carrying pNDM220, Δ*tacAT* pNDM220::*t8* (+TacT), and TS-*pth S*. Typhimurium 12023 strains. To facilitate comparisons, decreasing amounts of Δ*tacAT* pNDM220::*t8* (+TacT) tRNA were loaded, as indicated by a triangle above the gel. The smear of peptidyl-tRNA detected in the TS-*pth S*. Typhimurium strain is highlighted by a bracket on the gel.

(C) Effect of TacT on Pth in in vitro hydrolase activity assays. Substrate peptidyl-tRNA (lane 1) was treated with purified Pth (lane 2), purified wild-type toxin (lane 4) or a mix of Pth and TacT (lane 3). Ac-CoA was added to all samples.

(D) Exposure of tRNA molecules acetylated by TacT in cell free expression assays to Proteinase K treatment in vitro. All samples were supplemented with [14C]Ac-CoA. TacT-dependent acetylation was revealed by autoradiography (lower panels).

(E) Purified peptidyl-tRNA molecules (lane 1) subjected to in vitro hydrolysis by purified recombinant Pth (lane 2) or commercial Proteinase K (lane 3).

tRNA and peptidyl-tRNA molecules were separated on acid-urea polyacrylamide gel and revealed by methylene blue staining.

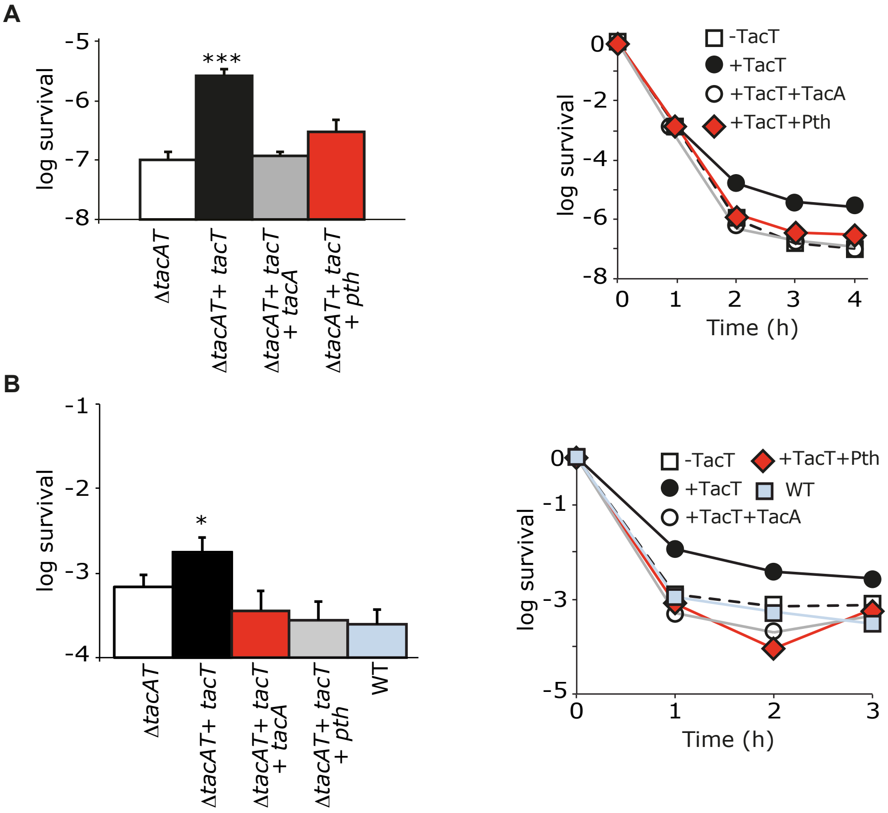

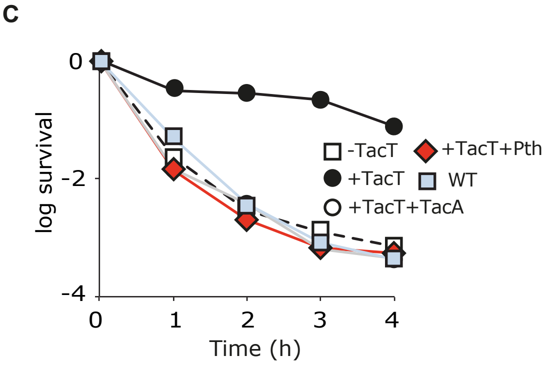

D

	WT	-TacT	+TacT
Gentamicin	12.5	12.5	12.5
Cefotaxime	12.5	12.5	12.5
Ciprofloxacin	2	2	2

Figure S7. TacT promotes persister formation (related to Figure 6)
(A) Proportion of bacteria surviving 4 h exposure (left panel) or representative killing curve (right panel) with bactericidal concentrations of gentamicin in cultures of *S*. Typhimurium 12023 Δ*tacAT*, Δ*tacAT* pNDM220::*tacT*, pNDM220::*tacT* and pBAD33::*tacA* or pNDM220::*tacT* and pBAD33::*pth*.
(B) Proportion of bacteria surviving 3 h exposure (left panel) or representative killing curve (right panel) with bactericidal concentrations of ciprofloxacin in cultures of *S*. Typhimurium 12023 Δ*tacAT*, Δ*tacAT* pNDM220::*tacT*, pNDM220::*tacT* and pBAD33::*tacA*, pNDM220::*tacT* and pBAD33::*pth* or WT.
(C) Representative killing curve with bactericidal concentrations of cefotaxime in cultures of *S*. Typhimurium 12023 Δ*tacAT*, Δ*tacAT* pNDM220::*tacT*, pNDM220::*tacT* and pBAD33::*tacA*, pNDM220::*tacT* and pBAD33::*pth* or WT.
Arabinose and IPTG were added to all cultures in fresh medium during lag phase then antibiotic treatment started 1 h later. Data represent the mean ± SEM (n≥3) and were analysed using a Student's t test (*p<0.05; ***p<0.001).
(D) MIC of antibiotics (ug/ml) for *S*. Typhimurium 12023 Δ*tacAT*, Δ*tacAT* pNDM220::*tacT* and WT. IPTG was added to all cultures throughout the assay for *tacT* expression induction.

S. Typhimurium 12023 strains		
Name	Description	Source or Reference
wild-type	12023 *S.* Typhimurium wild-type	NTCC
Δ*ta8* (then Δ*tacAT*)	12023Δ*ta8::kan*(STM4401-4402)	(Helaine et al., 2014)
Δ*parDE*	12023 *ΔparDE::kan*	(Helaine et al., 2014)
Δ*cobB*Δ*ta8*	12023 Δ*cobB::cat* Δ*ta8::kan*	This work
TS-*pth*	12023 pth_{G101D}*::kan*	This work

E. coli strains		
Name	Description	Source or Reference
PC2	BL21(DE3) *endA*::Tet^R T1^R pLysS	(Cherepanov, 2007)
BL21-AI	F-*omp*T*gal dcm ara*B::T7RNAP-*tet*A	Invitrogen

Plasmids		
Name	Description	Source or Reference
pNDM220	R1 *bla lacI^q* pA1/04/03	(Datsenko and Wanner, 2000; Gotfredsen and
pBAD33	p15 *cat araC* P_{BAD}	(Guzman et al., 1995)
pBAD18	pMB1 *bla araC* P_{BAD}	(Guzman et al., 1995)
pNDM220::*t8*	pNDM220 P*lac ::t8*	This work
pNDM220::*parE*	pNDM220 P*lac :parE*	This work
pBAD33::*a8*	pBAD33 P_{BAD}::*a8*	This work
pNDM220::*t8^{A93P}*	pNDM220 P*lac ::t8*^{A93P}	This work
pNDM220::*t8^{Y140F}*	pNDM220 P*lac ::t8*^{Y140F}	This work
pHISH::*t8^{Y140F}*	pET15b, *bla*, lacI^Q, T7::6His-T8^{Y140F}	This work
pRSFduet::*ta8*	pRSFduet-1, *kan*, lacI^Q, T7::6His-T8, T7::A8	Novagen, This work
pBAD33::*pth*	pBAD33 P_{BAD}::*pth*-6His	This work
pBAD18::*t8*	pBAD18 P_{BAD}::*t8*	This work
pNDM220::*t8^{R91E}*	pNDM220 P*lac ::t8*^{R91E}	This work
pNDM220::*t8^{K33E}*	pNDM220 P*lac ::t8*^{K33E}	This work

pNDM220::*t8*^{K36E}	pNDM220 P*lac* ::*t8*^{K36E}	This work
pNDM220::$^{t8R91E/K33E}$	pNDM220 P*lac* ::*t8*$^{R91E/K33E}$	This work
pNDM220::*t8*^{R77E}	pNDM220 P*lac* ::*t8*^{R77E}	This work
pNDM220::*t8*^{R78E}	pNDM220 P*lac* ::*t8*^{R78E}	This work
pNDM220::*t8*^{R158E}	pNDM220 P*lac* ::*t8*^{R158E}	This work
pNDM220::*t8*^{K146E}	pNDM220 P*lac* ::*t8*^{K146E}	This work

Table S1. List of strains and plasmids used in this study.

EXTENDED EXPERIMENTAL PROCEDURES

Bacterial mutagenesis. *S*. Typhimurium mutant strains were constructed using a one-step λ Red recombinase chromosomal inactivation system (Datsenko and Wanner, 2000). Plasmids pKD3 or pKD4 were used as templates to amplify the chloramphenicol (cat) or kanamycin (kan) resistance gene, respectively. Amplification reaction products were transformed into pKD46-containing bacteria expressing λ Red recombinase by electroporation (Datsenko and Wanner, 2000). Site directed mutagenesis was carried out using the QuickChange (Agilent) method on pNDM220::*t8* plasmid. Briefly, after amplification with appropriate primers (Table S1) to introduce point mutations, the plasmidic DNA was digested with DpnI. The digestion reaction was used to transform *E. coli* DH5α and plasmids purified from the clones obtained were verified by sequencing for presence of the desired point mutation.

Rates of protein, DNA and RNA synthesis

Cells were grown at 37 °C in LB to stationary phase over night. Cultures were then diluted to OD_{600} of 0.1 into fresh LB medium supplemented with 10 mM IPTG or the relevant antibiotic control, chloramphenicol (30 μg/ml), ciprofloxacin (1 μg/ml) or rifampicin (100 μg/ml) to inhibit protein synthesis, DNA synthesis and RNA synthesis respectively and were incubated at 37 °C with aeration. At 5, 30 and 60 min, 500 μl samples were harvested, normalized to an OD600 of 0.1 and incubated, at room

temperature, with 1 µCi of Methionine-^{35}S (protein synthesis), 2.5 of µCi Thymidine-2-^{14}C (DNA synthesis) or of 2.5 µCi Uracil-2-^{14}C (RNA synthesis). After 5 minutes of incorporation of radio-labeled isotopes, samples were chased for 10 min with 0.5 mg of the respective cold isotope. Cells were then washed with 500 µl 70% ethanol 3 times. Pellets were then resuspended in 10 µl 70% ethanol, dotted onto Whattman paper and exposed to a photostimulable phosphor (PSP) plate over night. Amounts of incorporated radioactivity were then visualized using a phosphorimager and quantified with Image J.

Expression and purification of recombinant T8^{Y140F}

Salmonella T8^{Y140F} sequence encoding residues were expressed as a C-terminal (His)6 fusion protein of the pHISH::*t8*^{Y140F} vector. *E. coli,* strain PC2, was grown in LB at 37 °C until an OD_{600} of 0.8 was reached, then protein expression was induced overnight at 18 °C by addition of 0.5 mM IPTG. Cells were lysed by sonication in 50 ml lysis buffer (50 mM Tris pH 7.5, 500 mM NaCl, 0.5 mM phenylmethanesulfonyl fluoride (PMSF)). Cleared lysate was incubated with 2 ml of NiNTA resin (Qiagen) for 1 hour at 4 °C with agitation. The lysate/resin mixture was applied to a column and washed with 50 ml wash buffer (50 mM Tris pH 7.5, 500 mM NaCl, 20 mM imidazole) and the protein eluted in 10 ml elution buffer (50 mM Tris pH7.5, 500 mM NaCl, 500 mM imidazole). The (His)6 tag was cleaved from the toxin with 3C protease. The protein was diluted to decrease the NaCl concentration to 100 mM and further purified by ion exchange column chromatography using a 1 ml HiTrap SP HP column (GE Healthcare) equilibrated with (50 mM Tris pH7.5, 100 mM NaCl, 10 mM β-mercaptoethanol). Elution was carried out using the Akta Prime protein purification system with a salt gradient ranging from 0.1 M to 1 M NaCl. Fractions containing the protein were pooled and subjected to size exclusion column chromatography using HiLoad Superdex-75 16/600 column (GE Healthcare) equilibrated with (50 mM Tris pH7.5, 150 mM NaCl, 10 mM β-mercaptoethanol).

Crystallization and structure determination of recombinant T8^{Y140F}

The purified T8Y140F protein at 10 mg/ml crystallized by hanging drop vapour diffusion against a reservoir of 22 % (w/v) Pentaerythritol ethoxylate (15/4 EO/OH), 100 mM Bis-tris pH6.5, 50 mM Ammonium sulphate, with crystalization enhanced by streak seeding. Crystals were harvested and frozen directly by immersion in liquid nitrogen. Diffraction data were acquired at Diamond light source (Oxfordshire, UK) on beamline I04-1. A high-resolution native dataset was integrated with mosflm (A.G.W. Leslie, 2007) and scaled using scala (Evans, 2006) of the ccp4i suite (Winn et al.,

2011). For experimental phasing, crystals were soaked in reservoir solution supplemented with 0.5 - 1 mM NaBr for up to 60 seconds before freezing. Highly redundant datasets were collected at the bromide edge from five NaBr-soaked crystals, processed using the XDS and XSCALE via the xia2 pipeline (Kabsch, 2010; Sauter et al., 2004; Winter et al., 2013; Z. Zhang, 2006) and merged together using blend (Foadi et al., 2013) to give a final dataset with >50 fold anomalous redundancy. Phases were obtained from single wavelength anomalous dispersion using PHENIX AutoSol, which also performed density modification and automated model building (Adams et al., 2010). The AutoSol output model was manually improved using coot (Emsley et al., 2010) and refined using phenix and refmac (Adams et al., 2010; Murshudov et al., 1997) against the high-resolution native data to give a final structure with an R_{free} of 22.5 % and good geometry (Table 1).

Suppression of T8 toxicity by overproduction of Pth

E. coli DH5α harbouring the pBAD18::*t8* plasmid were transformed with a pooled mixture containing the ASKA plasmid library (Kitagawa et al., 2005). After 1 hour recovery, the cells were plated on LB agar supplemented with the selective antibiotics, 50 mM arabinose and 250 μM IPTG to induce expression from both plasmids. Inserts of the ASKA library plasmids obtained from the recovered colonies were sequenced.

Expression and purification of recombinant Pth

Residues 1-194 of the Pth sequence from *Salmonella* 12023 strain were expressed with a C-terminal (His)6 purification tag from pBAD33 vector. Protein expression was induced in *Salmonella* or *E. coli* BL21-AI (Invitrogen) grown in LB supplemented with 0.2 % L-arabinose for 3 h at 37 ^{o}C. Cells were lysed in 300 mM NaCl, 50 mM Tris pH 7.5, 15 mM imidazole, 2 mM β-mercaptoethanol, with EDTA-free protease inhibitor cocktail (Roche Diagnostics). Cleared lysate was applied to TALON Cobalt resin (Clontech) and washed extensively firstly with lysis buffer and then with 150 mM NaCl, 50 mM Tris pH 7.5, 15 mM imidazole, 2 mM β-mercaptoethanol. Pth was eluted with 250 mM imidazole in 25 mM Tris pH 7.5, 150 mM NaCl, 2 mM β-mercaptoethanol, and further concentrated and dialysed in 25 mM Tris pH 7.5, 150 mM NaCl, 5 mM DTT. Pth purified from *Salmonella* strains co-expressing *t8* or not was analysed by nLC-ESI MS MS (BSRC Mass spectrometry and proteomics facility, University of St Andrews).

Analysis of tRNA and peptidyl-tRNAs by gel electrophoresis

Total RNA was extracted from *Salmonella* under acidic conditions to maintain the ester link between tRNA and amino acid/peptide as described in (Kohrer and Rajbhandary, 2008). This allows isolation of peptidyl-tRNA molecules carrying nascent chains of up to 80 residues (Janssen et al., 2012). Deacylated, aminoacyl- and peptidyl-tRNAs were separated by acid-urea PAGE as described in (Janssen et al., 2012) and stained by methylene blue (500 mM sodium acetate, 0.06 % methylene blue).

Pth *in vitro* functional assay

The activity of *Salmonella* Pth was assessed as previously reported in (Vandavasi et al., 2014). Briefly, purified peptidyl- or modified tRNAs were incubated with purified Pth (4 ug/ml) in activity assay buffer (10 mM Tris acetate, 10 mM magnesium acetate, 20 mM ammonium acetate pH 8.0) 1 h at 37 ^{o}C. The samples and uncleaved peptidyl-tRNA were run side-by-side on acid-urea PAGE and stained with methylene blue. Proteinase K (Qiagen) was used at 200 ug/ml in the same conditions.

Northern-blot

Northern analysis was used for detection of tRNA molecules. Total RNA was fractionated by acid/urea PAGE (10 %) and blotted onto a Nytran membrane (Whatman 0.45uM pore Nytran supercharge membrane) and hybridized with a single-stranded ^{32}P-labelled riboprobe primer complementary to specific tRNAs. The membrane was washed in SSC (Janssen et al., 2012).

Expression and purification of recombinant T8

Salmonella T8 (with an N-terminal (His)6 purification tag) and A8 sequence encoding residues were expressed from the dual expression vector pRSFduet::*ta8*. *E. coli* PC2 strain was grown in LB at 37 ^{o}C until an OD_{600} of 0.8 was reached, then protein expression was induced overnight at 18 ^{o}C by addition of 0.5 mM IPTG. Cells were lysed by sonication in 50 ml lysis buffer (50 mM Tris pH 7.5, 500 mM NaCl, 0.5 mM PMSF). Cleared lysate was incubated with 2 ml of NiNTA resin (Qiagen) for 1 hour at 4 °C with agitation. The lysate/resin mixture was applied to a column and washed with 50 ml wash buffer (50 mM Tris pH 7.5, 500 mM NaCl, 20 mM imidazole).

The TA complex bound to the column was denatured with 50 ml denaturation buffer (50 mM Tris pH 7.5, 500 mM NaCl, 5 M GnHCl) and then 50 ml of denaturation wash

buffer (50 mM Tris pH 7.5, 500 mM NaCl, 5 M GnHCl, 20 mM Imidazole). Finally the denatured toxin was eluted in 10 ml denaturation elution buffer (50 mM Tris pH 7.5, 500 mM NaCl, 5 M GnHCl, 500 mM Imidazole). The denatured protein was refolded via overnight dialysis at 4 °C in 2 l dialysis buffer (25 mM Tris pH 7.5, 25 mM NaCl, 5% glycerol) using 3500 MWCO dialysis tubing (Spectrum labs). Refolded T8 was concentrated and stored at -80 °C.

Cell free expression assay

In vitro cell free expression assays were carried out using the PURExpress *In Vitro* Protein Synthesis Kit (New England Biolabs [NEB], Massachusetts, USA), following the manufacturer's guidelines, with the exception of the addition of purified T8 (2 ng) supplemented or not with Ac-CoA (2 mM – Sigma Aldrich) or ^{14}C Ac-CoA (0.2 mM, 3.7 mBq - Perkin Elmer, Massachusetts, USA) where specified. Samples were analysed by SDS-PAGE or by acid/urea PAGE and stained by Coomassie, by autoradiography or by Northern blot.

Persister assays

The different bacterial strains were grown to stationary phase in M9 minimal medium overnight, they were then diluted to an OD_{600} of 0.05 into fresh M9 minimal medium, supplemented with antibiotics as appropriate. 1 mM IPTG and 0.2 % L-arabinose was used to induce expression of genes from pNDM220 and pBAD33 vectors respectively, and cultures were incubated at 37 °C. One hour after induction, samples were taken and CFU enumerated (t=0). Gentamicin (100 µg/ml) was added to the medium and cultures were incubated for 4 h at 37 °C. After the gentamicin treatment, 1 ml samples were collected and the surviving bacteria enumerated after the antibiotic was washed out (t=4).

References

A.G.W. Leslie, H.R.P. (2007). Processing Diffraction Data with Mosflm. Evolving Methods for Macromolecular Crystallography, 41-51.

Adams, P.D., Afonine, P.V., Bunkoczi, G., Chen, V.B., Davis, I.W., Echols, N., Headd, J.J., Hung, L.W., Kapral, G.J., Grosse-Kunstleve, R.W.*, et al.* (2010). PHENIX: a comprehensive Python-based system for macromolecular structure solution. Acta Crystallogr D Biol Crystallogr *66*, 213-221.

Cherepanov, P. (2007). LEDGF/p75 interacts with divergent lentiviral integrases and modulates their enzymatic activity in vitro. Nucleic Acids Res *35*, 113-124.

Datsenko, K.A., and Wanner, B.L. (2000). One-step inactivation of chromosomal genes in Escherichia coli K-12 using PCR products. Proc Natl Acad Sci U S A *97*, 6640-6645.

Emsley, P., Lohkamp, B., Scott, W.G., and Cowtan, K. (2010). Features and development of Coot. Acta Crystallogr D Biol Crystallogr *66*, 486-501.

Evans, P. (2006). Scaling and assessment of data quality. Acta Crystallogr D Biol Crystallogr *62*, 72-82.

Foadi, J., Aller, P., Alguel, Y., Cameron, A., Axford, D., Owen, R.L., Armour, W., Waterman, D.G., Iwata, S., and Evans, G. (2013). Clustering procedures for the optimal selection of data sets from multiple crystals in macromolecular crystallography. Acta Crystallogr D Biol Crystallogr *69*, 1617-1632.
Gotfredsen, M., and Gerdes, K. (1998). The Escherichia coli relBE genes belong to a new toxin-antitoxin gene family. Mol Microbiol *29*, 1065-1076.
Guzman, L.M., Belin, D., Carson, M.J., and Beckwith, J. (1995). Tight regulation, modulation, and high-level expression by vectors containing the arabinose PBAD promoter. J Bacteriol *177*, 4121-4130.
Helaine, S., Cheverton, A.M., Watson, K.G., Faure, L.M., Matthews, S.A., and Holden, D.W. (2014). Internalization of Salmonella by macrophages induces formation of nonreplicating persisters. Science *343*, 204-208.
Janssen, B.D., Diner, E.J., and Hayes, C.S. (2012). Analysis of aminoacyl- and peptidyl-tRNAs by gel electrophoresis. Methods Mol Biol *905*, 291-309.
Kabsch, W. (2010). Xds. Acta Crystallogr D Biol Crystallogr *66*, 125-132.
Kitagawa, M., Ara, T., Arifuzzaman, M., Ioka-Nakamichi, T., Inamoto, E., Toyonaga, H., and Mori, H. (2005). Complete set of ORF clones of Escherichia coli ASKA library (a complete set of E. coli K-12 ORF archive): unique resources for biological research. DNA Res *12*, 291-299.
Kohrer, C., and Rajbhandary, U.L. (2008). The many applications of acid urea polyacrylamide gel electrophoresis to studies of tRNAs and aminoacyl-tRNA synthetases. Methods *44*, 129-138.
Murshudov, G.N., Vagin, A.A., and Dodson, E.J. (1997). Refinement of macromolecular structures by the maximum-likelihood method. Acta Crystallogr D Biol Crystallogr *53*, 240-255.
Sauter, N.K., Grosse-Kunstleve, R.W., and Adams, P.D. (2004). Robust indexing for automatic data collection. J Appl Crystallogr *37*, 399-409.
Vandavasi, V., Taylor-Creel, K., McFeeters, R.L., Coates, L., and McFeeters, H. (2014). Recombinant production, crystallization and X-ray crystallographic structure determination of peptidyl-tRNA hydrolase from Salmonella typhimurium. Acta Crystallogr F Struct Biol Commun *70*, 872-877.
Winn, M.D., Ballard, C.C., Cowtan, K.D., Dodson, E.J., Emsley, P., Evans, P.R., Keegan, R.M., Krissinel, E.B., Leslie, A.G., McCoy, A*., et al.* (2011). Overview of the CCP4 suite and current developments. Acta Crystallogr D Biol Crystallogr *67*, 235-242.
Winter, G., Lobley, C.M., and Prince, S.M. (2013). Decision making in xia2. Acta Crystallogr D Biol Crystallogr *69*, 1260-1273.
Z. Zhang, N.K.S., H. van den Bedem, G. Snell and A. M. Deacon (2006). Automated diffraction image analysis and spot searching for high-throughput crystal screening. J Appl Cryst *39*, 112-119.
